# Anesthesia in the separation of conjoined twins (omphalopagus)—Example of a practical approach

**DOI:** 10.1007/s00101-023-01322-z

**Published:** 2023-08-11

**Authors:** Nicolas Leister, Sirin Yücetepe, Christoph Ulrichs, Christoph Menzel, Martin Dübbers, Angela Kribs, Bernd W. Böttiger, Uwe Trieschmann

**Affiliations:** 1 Department of Anesthesiology and Intensive Care Medicine, Faculty of Medicine and University Hospital Cologne, University of Cologne, Kerpener Straße 62, 50937 Cologne, Germany; 2 Pediatric Surgery, Department of General, Visceral, Cancer and Transplantation Surgery, Faculty of Medicine and University Hospital Cologne, University of Cologne, Cologne, Germany; 3 Department of Pediatrics, Medical Faculty and University Hospital Cologne, University of Cologne, Cologne, Germany

## Treten Sie in den Austausch

Diese Arbeit wurde für *Die Anaesthesiologie* in englischer Sprache eingereicht und angenommen. Wenn Sie Fragen zu dieser Kasuistik haben oder mehr wissen möchten, nehmen Sie bitte über die Korrespondenzadresse am Ende des Artikels Kontakt auf. Die Autorinnen und Autoren freuen sich auf den Austausch mit Ihnen.

While the total prevalence of conjoined twins is 1.47 per 100,000 births, the incidence of the appearance of isolated omphalopagus (joined at the umbilicus) is 5.5% with respect to all known variants [1, 2]. The overall mortality rate in liveborn conjoined twins is 61% and most deaths occur in the first 24–48 h [3]. The survival rate of isolated omphalopagus is described in the current literature as up to 80% [1]. Considering these facts it can be summarized that this case report represents a rare occurrence in the work as a pediatric anesthesiologist, with relatively good prospects of success, even if an expected 20% mortality is by no means part of the normal routine of pediatric anesthetists.

## The case

The twins were born by cesarean section at 28 weeks gestational age after a breech presentation in May 2022. Omphalopagus was evident. The combined birth weight was 2290 g, and both required continuous positive airway pressure (CPAP). Separation surgery was performed at a gestational age of 40 weeks in August 2022 at a combined weight of 5700 g.

## Involvement of pediatric anesthesiology

The first contact between the twins and the department of anesthesiology was soon after delivery, when the twins were in the period of respiratory stabilization with CPAP, initially applied by nasopharyngeal tubes and subsequently by nasal prongs. They could be stabilized without major problems, no infections and feeding was well tolerated. Therefore, the magnetic resonance imaging (MRI) for planning of the operation was delayed to week 31 and then discussed with all medical disciplines involved.

As airway management was considered the most critical challenge due to the fact that the twins were facing each other, the decision was made to install an intravenous (IV) line in each of the twins and to minimize the procedural sedation to a single dose of chloral hydrate before transport to the MRI suite. Even though the sedation dose was kept very low and thus respiratory depression seemed very unlikely, emergency equipment suitable for MRI was available for both patients. This modified feed and wrap technique led to successful imaging. Both children received separate injection of contrast agent showing that the circulation was separated.

If the children had become agitated and thus the MRI would not have been reasonably possible, the decision was to abandon imaging and proceed with a modification to deeper sedation and, if necessary, anesthesia on a subsequent day.

Weeks later, after the children had grown adequately and successful surgical separation became increasingly realistic, the decision to separate was made by an interdisciplinary consensus also with the parents.

## Preparations of pediatric anesthesiologists on the anesthesia for separation

Several interdisciplinary meetings for perioperative and intraoperative procedure planning took place in the weeks before the scheduled surgery date. The pediatric anesthesiology team decided that 4 senior physicians (2 for each child) and 2 nurses, all with profound and long-term pediatric expertise would care for the 2 children during separation. The preanesthesia assessment was combined with a positioning rehearsal regarding the possibilities for airway management and sonography of the great vessels with respect to arterial and venous cannulation. As positioning one twin on top of the other for intubation requires repositioning of the twins at least twice, the decision was made to manage the airway in the lateral position.

**Fig. 1:**
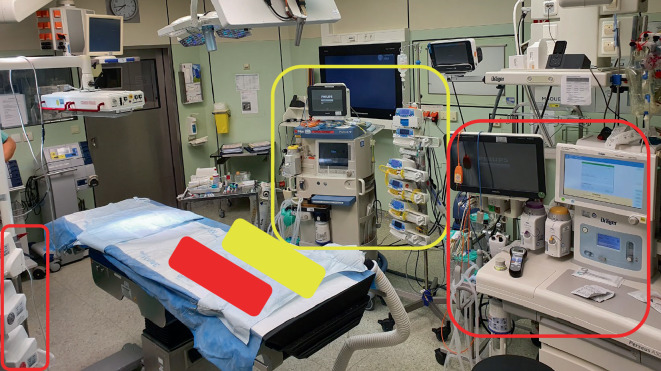
Photograph of the operating room during the simulation trial showing the positioning of equipment; *red*: position of equipment patient I; *yellow*: position of equipment patient II

A simulation trial with all the required anesthesiological and surgical equipment took place 2 days before the planned separation in the designated operating room (Fig. 1). The exact planning of the procedure included two anesthesia machines, one with the ability to be moved after the separation (long tubing equipment), two sets of perfusion pumps (again one with long tubing), color-coded infusion lines and syringes, exact procedure for induction of anesthesia and repositioning after separation. Due to limited space and sterile surgical zones, the team decided against positioning both anesthesia machines at the head end of the table (one on the left and one on the right) and to fix the monitoring and syringe pumps mirror inverted. The required wall connections (e.g., oxygen and gas exhaust) were available in duplicate in the chosen operating room. In addition, it was decided to reduce the number of major procedures on pediatric patients in the hospital for the day in question in order to prevent the capacities of the intensive care unit (ICU) from being exceeded.

## Performing anesthesia

**Fig. 2:**
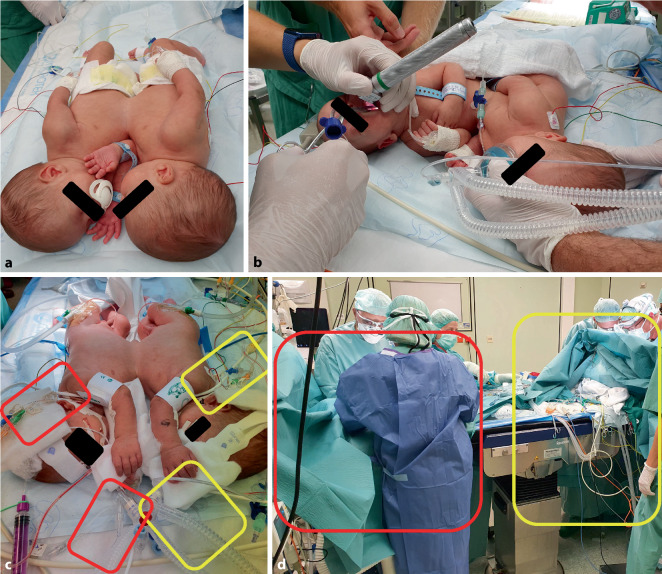
**a** Omphalopagus at top of adult operating table. **b** Intubation of the left twin (I). **c** Status after anesthesia induction: position of i.v. lines and ventilation tubes (*red*: I/*yellow*: II). **d** Position after separation: (*red*: I/*yellow*: II)

The children were placed at the top of a normal adult operating table (Fig. 2a). Inhalational induction was performed (despite an intravenous line being in place) to maintain spontaneous breathing and to allow an incremental transition to mask ventilation. The drugs for an intravenous induction were prepared in case a straightforward strategy would have become necessary.

The left child (I) was the first to be started. After administration of the opioids and the muscle relaxant, the right twin (II) also showed signs of respiratory insufficiency requiring assisted mask ventilation. The airways were secured successively, both in the first attempt by conventional means, with 2 video laryngoscopes on standby (Fig. 2b). The decision to use conventional intubation was made by consensus among the team, due to the limited space between the children’s heads. Subsequently, one after the other, an arterial cannula (brachial artery) and a central venous catheter (jugular vein) were positioned into the vessels located above under sonographic view in both children (Fig. 2c). Preplanning and prior sonographic selection of the vessels to be punctured proved to be of significant benefit to the patients (e.g., radial arteries were found to be too small for successful puncture). Near-infrared spectroscopy was used in both children. Crossing the midline between the children by wires, lines or tubes was consequently avoided. In both anesthesia teams, there was a clear assignment of the senior physicians involved with respect to invasive and organizational activities. Although the anesthesiology team was large, this ensured a smooth workflow, including on-time documentation.

Intraoperatively, it was found that apart from connective tissue, there was a small liver bridge between the twins. After the final separation, the right twin (II) was moved to the end of the operating table (Fig. 2d). During the transfer, it was apparent that the prior precise planning of the procedures and the positioning trial prevented confusion in the area of cables and infusion lines. After final abdominal closure in both children, they could be transferred to the ICU in stable circulatory condition without catecholamine support.

## Learning points

- The anesthesiological management of omphalopagus separation is demanding, even though the surgical steps are usually not exaggerated compared to other conjoined twin variants.
- Precise preoperative planning, simulation and interdisciplinary collaboration at all levels (medical professional, administrative, etc.) are mandatory.
- Ultrasound imaging of the vascular situation during the anesthesiological examination facilitates the selection of suitable puncture sites and allows the puncture sequence to be planned well in advance.
